# Discovery of Arthritis in Psoriasis Patients for Early Rheumatological Referral (DAPPER): Protocol for a Longitudinal Observational Study

**DOI:** 10.2196/31647

**Published:** 2021-11-16

**Authors:** Tamara W van Hal, Juul MPA Van den Reek, Hans MM Groenewoud, Marcel C Pasch, Frank HJ Van den Hoogen, Mark H Wenink, Elke MGJ De Jong

**Affiliations:** 1 Department of Rheumatology Sint Maartenskliniek Nijmegen Netherlands; 2 Department of Dermatology Radboud University Medical Center Nijmegen Netherlands; 3 Radboud Institute for Health Sciences Radboud University Medical Center Nijmegen Netherlands; 4 Faculty of Medical Sciences Radboud University Nijmegen Netherlands

**Keywords:** psoriasis, psoriatic arthritis, screening

## Abstract

**Background:**

One in three patients with psoriasis will develop psoriatic arthritis (PsA). If left untreated, this can lead to pain, impaired function, and irreversible joint damage. Timely recognition and referral to a rheumatologist are therefore key. However, current methods used to screen patients with psoriasis for those who might benefit from referral to a rheumatologist are not performing well enough.

**Objective:**

The Discovery of Arthritis in Psoriasis Patients for Early Rheumatological Referral (DAPPER) study is designed to determine the prevalence of PsA in a psoriasis population and to find parameters that can be used to develop a new or enhance an existing instrument for a rheumatological referral.

**Methods:**

DAPPER is a longitudinal observational study with a 1-year follow-up. Patients with psoriasis (N=300) who are treated at an outpatient dermatological clinic will be screened extensively for signs and symptoms of PsA by a trained rheumatologist. If there is clinical suspicion of PsA and the patient is not yet treated by a rheumatologist, referral to the Department of Rheumatology will follow for confirmation of the diagnosis and further care. After 1 year, data on changes in quality of life and PsA and psoriasis disease activity will be collected from the referred patients. The screening visit will be used to gather demographical and medical data, which can later be used to develop the aforementioned screening instrument.

**Results:**

Inclusion started in June 2019 and finished in June 2021. Follow-up with newly discovered patients with PsA is ongoing.

**Conclusions:**

The DAPPER study is specifically designed to improve the detection of existing PsA in a dermatologic outpatient setting. Although internal validity will be tested, external validity will have to be checked using a second validation cohort. To predict the development of PsA in the future, longitudinal/prospective data collection is required and will be performed in a follow-up study (DAPPER-i).

**Trial Registration:**

Dutch Trial Register NTR7604; https://www.trialregister.nl/trial/7397

**International Registered Report Identifier (IRRID):**

DERR1-10.2196/31647

## Introduction

Psoriasis is a common immune-mediated skin disease. Besides skin and nails, psoriatic disease can also involve several other domains such as the entheses and the peripheral as well as the axial joints. This involvement of the musculoskeletal system defines psoriatic arthritis (PsA). PsA is an inflammatory rheumatic disease, related to other spondyloarthritides such as reactive arthritis, ankylosing spondylitis, or inflammatory bowel disease–associated arthritis. About one in three patients with psoriasis in the dermatological outpatient clinic will eventually develop PsA [1,2]. The order and amount of domains involved display a large variation in different patients and at different time points [3]. However, the musculoskeletal symptoms often develop after the disease shows itself in skin or nails. On average, the lag time between skin and joint involvement is 10 years [4].

When joints or entheses become inflamed, these can cause significant pain and have a large impact on the quality of life [5]. Moreover, ongoing inflammation of joints can lead to irreversible joint damage and disability [6,7]. Early and adequate treatment of arthritis leads to an improvement of both joint function and quality of life [8,9]. Therefore, it is important to recognize and treat patients with concomitant arthritis as soon as possible.

The treatment strategies for psoriasis and PsA show considerable overlap [10,11]. Several pharmacological options are effective and recommended to treat both skin and joints. These encompass, for example, conventional systemic drugs such as methotrexate and several biological drugs such as tumor necrosis factor alpha inhibitors and interleukin-17 inhibitors. However, some options are only available for one of these disease entities. This may be because of the delivery mode (eg, topical application of creams for psoriasis or local injections of corticosteroids for PsA) or because of a difference in efficacy in controlling either joint or skin disease (eg, retinoids for psoriasis and leflunomide for PsA). This could mean that the therapy a patient uses for their skin can also be effective for their musculoskeletal complaints.

To ensure early adequate treatment and prevent (irreversible) morbidity, early recognition and early referral to a rheumatologist are key. The combined guidelines of the American Association of Dermatologist and the National Psoriasis Foundation calls screening of patients with psoriasis for PsA “essential at each visit” [12]. However, recognition of inflammatory joint complaints is not part of the dermatological scope. In addition, due to a large prevalence of noninflammatory joint complaints, referral of all patients with musculoskeletal pain is considered an unnecessary drain of resources. Therefore, about one in three patients with PsA remain unrecognized in the dermatological clinic [1] and are at risk for irreversible damage.

To aid the recognition of PsA by dermatologists, several screening questionnaires have been developed [13-16]. Most of these are based on multiple patient-reported signs or symptoms and result in a cumulative score. Referral to a rheumatologist is recommended when a certain score is reached. Unfortunately, testing of these questionnaires in new cohorts often had disappointing results [17,18]. The long average lag time between psoriasis and PsA also necessitates repeated use of a screening tool on a regular basis. However, none of the questionnaires were validated for reuse. These are all clues that current referral strategies are inadequate.

By screening a psoriasis population for the presence of concomitant PsA, we want to determine the prevalence of (undiscovered) PsA in this group. During this screening visit, we will gather data about several clinical characteristics. These will be used to ultimately develop a new or enhance an existing instrument for rheumatologic referral. This study is therefore called the Discovery of Arthritis in Psoriasis Patients for Early Rheumatology Referral (DAPPER).

## Methods

### Aim

The aim of this study is to determine the number of patients with (untreated) PsA in a group of psoriasis patients in a dermatological outpatient clinic. Furthermore, we want to optimize the detection of PsA in patients with psoriasis in a dermatological outpatient clinic. For this purpose, we defined the following research questions.

The primary objective is to determine the prevalence of very early, newly-discovered, and known PsA in a cohort of patients with psoriasis treated at a dermatology outpatient clinic.

The secondary objectives include determining if, in newly diagnosed patients with PsA, PsA disease activity and quality of life differ before and 1 year after rheumatological referral in case of PsA; discovering clinical parameters that are associated with the presence of PsA in a cohort of patients with psoriasis; and using the aforementioned parameters to develop a new or enhance an existing screening tool for concomitant PsA in patients with psoriasis.

### Design

The DAPPER study is a monocenter observational study with a follow-up of 1 year. We will examine 300 patients stratified 1:1:1 according to current dermatological treatment (topical or UV therapy only, conventional systemic medication but no biologicals, biological therapy).

The initial screening at the dermatology department will include a 68 tender joint count, 66 swollen joint count, a dactylitis count (0-20), and enthesitis scores (Leeds Enthesitis Index [LEI] [19] and the enthesitis score of the Spondyloarthritis Research Consortium of Canada [SPARCC] [20]). Inflammatory back pain will be assessed via the criteria of the Assessment of Spondyloarthritis International Society [21]. At this study visit, no laboratory tests or imaging will be performed for diagnostic purposes.

To investigate possible identifying characteristics or confounders for the detection of PsA, the study visit will also be used to gather demographical data (comorbidity, treatment data, and clinical characteristics of the skin). An example of the interview guide used is shown in Multimedia Appendix 1.

#### Referral and Referral Criteria

If there is a clinical suspicion of PsA in the study visit according to the study physician (trained rheumatologist), the patient will be referred to the Department of Rheumatology. Referral to a rheumatologist will be at the discretion of the investigator. A patient will be referred when not under current rheumatological care and when meeting one of the following criteria: one or more swollen joints, clinical evidence of inflammatory enthesitis, and/or inflammatory back pain. Other reasons to suspect PsA can also give rise to referral (eg, restricted movement in a joint or prolonged morning stiffness). From there on, these patients will be investigated and treated as in regular PsA care. This will include confirmation of the diagnosis with additional laboratory tests and imaging, and treat-to-target via the Psoriatic Arthritis Disease Activity Score (PASDAS) [22].

#### Follow-up

Only those patients with a newly discovered PsA, as confirmed by a rheumatologist after referral, will be approached for follow-up after 1 year. At that moment, changes in treatment, disease activity, and health-related quality of life (HR-QoL) will be noted.

### Study Setting

This study will be carried out in the outpatient clinic of the Department of Dermatology in an academic center in the Netherlands (Radboud University Medical Center, Nijmegen). This department is a national psoriasis expertise center. Patients will initially be screened at the Department of Dermatology for signs or symptoms of enthesitis, dactylitis, arthritis, or inflammatory back pain by a trained rheumatologist. When additional rheumatological evaluation is required, patients are preferentially referred to the Department of Rheumatology of the Sint Maartenskliniek in Nijmegen. Here, the patient will be assessed by a rheumatologist with special expertise in PsA. When requested by the patient, a referral to another rheumatologic center is also possible.

### Participants

All patients with a clinical diagnosis of psoriasis who are treated at the outpatient clinic are eligible for this study. Neither current nor previous treatment by a rheumatologist nor a previous diagnosis of PsA are exclusion criteria. Patients must be 18 years or older and be able to give written informed consent.

#### Study Size

For the logistic model, we aim to use 5 to 10 independent variables. The number of independent variables used in the model will be restricted to 1 per 10 events (ie, 1 per 10 PsA cases). Therefore, we aim to have 50 to 100 PsA cases. Assuming a prevalence of PsA of 20% to 30% [1], this means we need 167 (prevalence 30%, 5 predictors) to 500 (prevalence 20%, 10 predictors) patients with psoriasis. Using a total number of 300 patients, we expect to find up to 60 to 90 PsA cases, ensuring we can incorporate 6 to 9 independent variables.

#### Recruitment

All patients eligible for the study will be asked for study participation by their dermatologist. Written and oral information about the study will be given by the investigator. A study visit will be planned adjacent to a regular outpatient visit with the dermatologist. Before the study visit starts, written informed consent is obtained from the patients.

### Outcome Measures

The primary outcome measure will be the percentage of investigated patients with the diagnosis of PsA. This diagnosis will be accepted if it was confirmed by a rheumatologist in correspondence. Fulfillment of Classification Criteria for Psoriatic Arthritis (CASPAR) is not required [23]. After 1 year, patient files of the referred patients will be checked to confirm the diagnosis. If the suspicion of active PsA is confirmed, treatment changes and their effect on disease activity will be noted. Alternatively, the other rheumatological diagnosis will be noted.

In the referred patients with PsA, HR-QoL will be assessed via two disease-specific questionnaires at referral and 1 year thereafter. Skin-related impact will be explored via the Dermatological Life Quality Index (DLQI) [24]. Joint-related impact will be explored via the Psoriatic Arthritis Impact of Disease (PsAID) [25].

### Outcome Variables

#### Prevalence of PsA

To ascertain the presence of PsA, we will ask the patient about joint and enthesis complaints (location, pattern, and intensity), morning stiffness (duration), and whether or not they ever had a diagnosis of arthritis. For confirmation of arthritis, dactylitis, or enthesitis, we will perform joint counts (swollen, tender, and dactylitis) and enthesitis indices (LEI and SPARCC). After referral, the diagnosis of PsA or alternative diagnosis will be retrieved from (the correspondence gathered in) the electronic patient file.

#### Effect of Referral

In referred patients with confirmed PsA, we will retrieve data at time of referral and 1 year later. We will use the PASDAS as a disease activity score, which gives a full overview of the PsA disease spectrum. We will evaluate both the combined disease activity score and the specific scores of tender and/or swollen joints, dactylitis, and enthesitis. In addition, treatment changes (either instigated by rheumatologist or dermatologist) will be retrieved from the electronic patient file. Impact on HR-QoL will be assessed by questionnaires before referral and after 1 year (DLQI [24], PsAID12 [25]).

#### Possible Identifying Characteristics for the Presence of PsA in Psoriasis

We will gather information about demographic variables, comorbidity, intoxications, and family history. Family history and comorbidity will be targeted at diseases that are associated with spondyloarthritis, such as uveitis, psoriasis, and inflammatory bowel disease. Next to that, the Charlson Comorbidity Index [26] and Functional Comorbidity Index [27] will be used to evaluate a total comorbidity burden. Data about comorbidity specifically associated with either psoriasis or PsA (eg, hepatic [28], psychological [29], and cardiovascular [30] diseases) will be added. In addition, current and previous treatment for either PsA or psoriasis will be noted. Severity and location of psoriasis (via Psoriasis Area and Severity Index [31] and body surface amount will be noted. Nail involvement will be assessed via Nail Psoriasis Severity Index [32] and Nijmegen Nail Psoriasis Activity Index Tool [33]. Three of the currently used screening questionnaires (ie, Psoriasis Epidemiology Screening Tool [PEST], Toronto Psoriatic Arthritis Screen [ToPAS], and Psoriatic Arthritis Screening and Evaluation [PASE]) will be used to collect clinical characteristics that have been previously discovered in their development [13-15].

### Statistics

#### Prevalence

The primary outcome of this study will be the point prevalence (number per 100 patients) of PsA in established patients with psoriasis. Sensitivity analyses will be performed by including or excluding patients with an uncertain diagnosis after 1 year, patients who refuse referral, or patients who are otherwise lost to follow-up.

#### Effect of Referral

The effect of referral on treatment changes, disease activity, and HR-QoL will be assessed qualitatively in an explorative descriptive matter. No formal statistical analyses will be applied.

#### Possible Identifying Characteristics for the Presence of PsA in Psoriasis

The identifying value of various clinical markers for the presence of PsA in psoriasis will be processed as independent variables in a univariate logistic regression model. Diagnosis of PsA (yes/no) will be the dependent variable. Variables that are statistically related to the outcome (*P*≤.20 in univariate modeling) and are clinically and methodologically feasible (based on a favorable balance between prevalence in the cohort, effect size, and ease of measurement) will be selected. The subsequent selection of variables will be tested in a multivariable logistic regression model with backward stepwise selection. Sensitivity analysis will be performed by reclassifying patients with an uncertain diagnosis as cases. The number of possible independent variables will be limited based on a minimum of 10 events (PsA diagnoses) per variable. Bootstrapping will be used to assess the internal validity of the model in terms of overoptimism and shrinkage.

### Data Handling

The collected data will be entered in CASTOR, an electronic database set up for clinical trials. Data will be coded and kept by personnel trained in Good Clinical Practice. Handling of personal data will comply with the Data Protection Law.

During the informed consent procedure, patients will be asked if gathered data can be used for further research involving psoriasis or PsA. Only data from patients who gave consent for this can be reused in accordance to FAIR (Findable, Accessible, Interoperable, Reusable) principles.

Monitoring will be performed by certified personnel from the Radboud University Medical Center, according to the guidelines of the NFU (Dutch Federation of University Medical Centers).

### Ethical Considerations

DAPPER has been approved by the Ethical Committee of the region Arnhem-Nijmegen, Radboud University Medical Centre (NL68137.091.18). It has been registered in the Dutch Trial Register (NTR 7604). All study procedures will be performed in accordance with the ICH guidelines on Good Clinical Practice and the principles of the Declaration of Helsinki.

## Results

Ethical approval was obtained by the Ethical Committee of the region Arnhem-Nijmegen, Radboud University Medical Centre (NL68137.091.18) in April 2019. Inclusion started in June 2019 and finished in June 2021. Follow-up is expected to be finished in December 2022, thereby ending the study.

## Discussion

PsA is an inflammatory disease of joints and entheses, which can cause pain, disability, and a diminished quality of life. Moreover, prolonged arthritis can lead to permanent irreversible joint damage [6,7]. Early recognition, for example, by screening populations at high risk for PsA, may be able to prevent joint damage by facilitating timely treatment. The high prevalence of PsA in patients with psoriasis and the fact that skin complaints mostly appear years before joint involvement make this population suitable for the implementation of screening. However, current screening questionnaires are not sufficient. Therefore, we wish to determine if current screening and referral strategies are satisfactory, and to improve them if necessary.

In our study, we used three of the previously developed questionnaires: PASE, PEST, and ToPAS [13-15]. Although their sensitivity and specificity could be improved, we feel that the possibly identifying variables used in these questionnaires warrant further evaluation [17,18]. Our study has several strengths that may overcome the suboptimal performance of the beforementioned questionnaires. First, the PASE and PEST development studies were hampered by a low amount of PsA cases (17 and 12, respectively) [13,14]. Second, the setting of our study in the Dermatology Department ensures access to the target population with minimal extra burden for the patient. Although the ToPAS study included 164 patients with PsA, most of these were recruited via the Rheumatology Department. Only 123 study participants were recruited via the Dermatology Department, giving rise to 30 PsA cases [15]. As stated in the study size, we expect to find 60 to 90 PsA cases in our cohort. Therefore, we expect our model to be more precise.

To develop a good referral tool, the patient population on which development of the model is based is crucial. A limitation of our study could be the academic setting. However, to ensure a more representative case mix, we stratified for current treatment. By using treatment modality as a proxy for severity and by limiting the amount of patients using third-line therapy (eg, biological and targeted therapies), we aim to simulate a population representative of an average dermatological outpatient clinic. Noteworthy in this context is the fact that this study does not provide a validation cohort. Internal validity will be checked by bootstrapping. Before implementing the referral tool, external validity has to be assessed via a second (validation) cohort. Ideally, this second cohort will be found at one or more other centers, both academic and nonacademic.

A second important choice is the definition of the outcome. In this cohort, we choose not to use the CASPAR criteria [23]. These classification criteria are designed to ensure a homogenous PsA population at the start of the trial. However, these criteria are not meant to be used as diagnostic criteria. In clinical practice, the diagnosis made by the rheumatologist (expert opinion) remains the gold standard. However, since all referred patients in this cohort will have clinical psoriasis, they only need one more point (ie, nail psoriasis, negative rheumatoid factor, dactylitis, or PsA-specific lesions on imaging) to fulfill the criteria (assuming that there is an inflammatory joint or entheseal lesion). Therefore, we expect that (almost) all patients diagnosed with PsA from this cohort will fulfill CASPAR criteria.

The long lag time between skin and joint involvement (on average, 10 years [4]) also has several consequences for a referral tool. When screening for current concomitant PsA, a tool must be applied several times during follow-up. Ideally, every contact moment between treating dermatologist and patient would be an opportunity to check for suspicion of PsA. This means that the investment to use the tool must be minimal, both in time and in money. Therefore, we choose to use only clinical parameters in our data collection. It will be easy for a dermatologist to gather this data from a patient without the necessity for further laboratory or imaging techniques.

A second consequence of the repeated use of the referral tool is that its validity in reuse must be evaluated. With this study design, we cannot assess this validity in repeated use. Implementation of the developed tool in the follow-up of this cohort can be a way to test this.

Ideally, one would want to predict the development of PsA before symptoms or damage arise. However, it is important to realize that the aforementioned design of the DAPPER is focused on *detection* rather than *prediction*. We believe that prediction is a much desired goal, and several studies have reported about signs and symptoms that may present themselves some time before development of full-blown PsA [34,35]. However, the long lag time of PsA in patients with psoriasis means that development and validation of a prediction tool takes a decade or longer. Therefore, we choose to focus on improving the detection of PsA until such prediction tools are available.

In conclusion, the DAPPER study will help improve psoriasis care by providing us information about the extent of (un)diagnosed arthritis in this population. The gathered data about the patients with and without arthritis can then be used to develop an improved screening and referral tool to ensure adequate and timely care for those patients who need it.

## Appendix

Multimedia Appendix 1 Interview guide.

## Abbreviations

CASPAR: Classification Criteria for Psoriatic Arthritis
DAPPER: Discovery of Arthritis in Psoriasis Patients for Early Rheumatology Referral
DLQI: Dermatological Life Quality Index
FAIR: Findable, Accessible, Interoperable, Reusable
HR-QoL: health-related quality of life
LEI: Leeds Enthesitis Index
NFU: Dutch Federation of University Medical Centers
PASDAS: Psoriatic Arthritis Disease Activity Score
PASE: Psoriatic Arthritis Screening and Evaluation
PEST: Psoriasis Epidemiology Screening Tool
PsA: psoriatic arthritis
PsAID: Psoriatic Arthritis Impact of Disease
SPARCC: Spondyloarthritis Research Consortium of Canada
ToPAS: Toronto Psoriatic Arthritis Screen
