# Supplementary material for: Discovery of Arthritis in Psoriasis Patients for Early Rheumatological Referral (DAPPER): Protocol for a Longitudinal Observational Study
Source: JMIR Res Protoc. 2021 Nov 16;10(11):e31647. doi: 10.2196/31647 (PMC8663491; doi:10.2196/31647)
Supplement: Multimedia Appendix 1 [file resprot_v10i11e31647_app1.docx]

**DAPPER
Interview guide**

**Demographic data:**
1a. Year of birth
1b. Age
1c. Sex

**Intoxications:**
2. Smoking: currently / in the past / never
If applicable:
2a. Year of start smoking
2b. Year of stop smoking
2c. Number of cigarettes per day
3 Use of alcohol: currently / in the past / never
2a. If currently: amount per day

**Family history for SpA-related diseases (to second degree)**
If yes, note relationship to patient
4a. Psoriasis
4b. Psoriatic arthritis
4c. Inflammatory bowel disease
4d. Uveitis
4e. Axial spondyloarthritis

**Comorbidity**Charlson Comordity Index^26^, Functional Comorbidity Index^27^
Supplemented with:
5. Known diagnosis of PsA
If yes, note date of diagnosis
6. Diseases associated with PsA and/or Pso
If yes, note year of diagnosis:
6a. Hypertension
6b. Hypercholesterolemia
6c. Thyroid disease
6d. Cholelithiasis, cholangitis or cholecystectomy
6e. Celiac disease
6f. Obesity
6g. Bariatric surgery
7. Other SpA-related diseases
7a. Uveitis
7b. Inflammatory bowel disease
7c. Axial spondyloarthritis
8. Diseases with impact on possible treatments
8a. Hepatitis B infection
8b. Hepatitis C infection
8c. Hepatic steatosis
8d. Tuberculosis
8e. Eczema/atopic dermatitis
8f. Hidradenitis suppurativa
9 Other rheumatologic disease with impact on possible symptoms
9a. Fibromyalgia
9b. Gout

**Physical exposure during occupational or leisure activities**10a. Is the current occupation physical demanding?
10b. Sports injury in the past year?
10c. Fall or other accidental trauma in the past year?
10d. Fracture (which year)?

**Topical medication for Pso:**Note first and last year of use, if known
11a. Corticosteroid ointment: currently/in the past/never
11b. Vitamin D creams: currently/in the past/never
11c. Calcineurin inhibitor creams: currently/in the past/never
Note first and last year of use and number of courses, if known
11d. Dithranol/cignolin creams
11e. UVB phototherapy
11f. (P)UVA phototherapy

**Systemic medication for Pso or PsA:**
For example, but not limited to: methotrexate, fumaric acid, leflunomide, biologicals.
12a. Name of medication
12b. Year of start
12c. Physician who started it (dermatologist/rheumatologist/other)
12d. Year of discontinuation
12e. Physician who discontinued medication
12f. Reason for discontinuation (e.g. primary or secondary ineffectiveness, pregnancy or pregnancy wish, side effects, contra-indication, other)
12g. Highest dose/shortest interval
12h. Currently used dose/interval

**Other medication with a possible effect on Pso or PsA:**
Note last known date of use
13a. NSAID
13b. Prednisone (plus route of administration, e.g. oral, intramuscular, intra-articular)
13c. Lithium
13d. Beta-blocker
13e. ACE-inhibitor
13f. Tetracycline
13g. Terbinafine
13h. Immunomodulators (e.g. cancer treatment)

**Screening questionnaires for PsA in Pso**
PEST^13^, Topas^15^, EARP^16^

**Characteristics of skin involvement**
14a. Year of psoriasis initiation
14b. Locations involved (at start, during disease, during last year; scalp, face, extremities, trunk, inversa, genital, palmoplantar, nails)
14c. Morphology involved (at start, during disease, during last year; plaque, guttate, pustulosa, erythroderma)
14d. VAS of skin involvement severity
14e. Koebner-phenomenon

**Characteristics of nail involvement**
14a. Pitting (never, more than one year ago, last year, currently)
14b. Oil drop phenomenon (never, more than one year ago, last year, currently)
14c. Leukonychia (never, more than one year ago, last year, currently)
14d. Distal onycholysis (never, more than one year ago, last year, currently)
14e. Crumbling (never, more than one year ago, last year, currently)
14f. Red spots in lunula (never, more than one year ago, last year, currently)
14g. Splinter hemorrhages (never, more than one year ago, last year, currently)

**Characteristics of joint involvement**
Pain
15a. Joint pain and location
15b. Time of day with worst complaints (night, morning, afternoon, evening)
15c. Worsening or improvement on exertion
15d. VAS on joint involvement
Swelling
16a. Joint swelling and location of swelling
16b. Rubor, calor of joints
16c. Swelling of Achilles tendon
Back pain
Inflammatory back pain according to ASAS criteria^21^
Other
17a. Morning stiffness: how long, change in the last year
17b. Tiredness: VAS, change in the last year

ACE = angiotensin converting enzyme; ASAS = Assessment of SpondyloArthritis International Society; NSAID = non-steroidal anti-inflammatory drug; PsA = psoriatic arthritis; Pso = psoriasis; PUVA = psoralen-UVA; SpA = spondyloarthritis; VAS = visual analogue scale
